# An *in vitro* anti-inflammatory effect of Thai propolis in human dental pulp cells

**DOI:** 10.1590/1678-7757-2023-0006

**Published:** 2023-06-05

**Authors:** Nutthapong KANTRONG, Jittranut KUMTAWEE, Teerasak DAMRONGRUNGRUANG, Subin PUASIRI, Anupong MAKEUDOM, Suttichai KRISANAPRAKORNKIT, Pattama CHAILERTVANITKUL

**Affiliations:** 1 Khon Kaen University Faculty of Dentistry Department of Restorative Dentistry Khon Kaen Thailand Khon Kaen University, Faculty of Dentistry, Department of Restorative Dentistry, Khon Kaen, Thailand.; 2 Khon Kaen University Faculty of Dentistry Department of Oral and Biomedical Sciences Khon Kaen Thailand Khon Kaen University, Faculty of Dentistry, Department of Oral and Biomedical Sciences, Khon Kaen, Thailand.; 3 Khon Kaen University Faculty of Dentistry Department of Preventive Dentistry Khon Kaen Thailand Khon Kaen University, Faculty of Dentistry, Department of Preventive Dentistry, Khon Kaen, Thailand.; 4 Mae Fah Luang University School of Dentistry Chiang Rai Thailand Mae Fah Luang University, School of Dentistry, Chiang Rai, Thailand.; 5 Chiang Mai University Faculty of Dentistry Department of Oral Biology and Diagnostic Sciences Chiang Mai Thailand Chiang Mai University, Faculty of Dentistry, Department of Oral Biology and Diagnostic Sciences, Center of Excellence in Oral and Maxillofacial Biology, Chiang Mai, Thailand.

**Keywords:** Cyclooxygenase-2, Dental pulp cells, Interleukin-1, Propolis, Prostaglandin E2

## Abstract

**Objective:**

To explore the potential for development of Thai propolis extract as a pulp capping agent to suppress pulpal inflammation from dental pulp infections. This study aimed to examine the anti-inflammatory effect of the propolis extract on the arachidonic acid pathway, activated by interleukin (IL)-1β, in cultured human dental pulp cells.

**Methodology:**

Dental pulp cells, isolated from three freshly extracted third molars, were first characterized for their mesenchymal origin and treated with 10 ng/ml of IL-1β in the presence or absence of non-toxic concentrations of the extract from 0.08 to 1.25 mg/ml, as determined by the PrestoBlue cytotoxic assay. Total RNA was harvested and analyzed for mRNA expressions of 5-lipoxygenase (5-LOX) and cyclooxygenase-2 (COX-2). Western blot hybridization was performed to investigate COX-2 protein expression. Culture supernatants were assayed for released prostaglandin E2 levels. Immunofluorescence was conducted to determine involvement of nuclear factor-kappaB (NF-kB) in the inhibitory effect of the extract.

**Results:**

Stimulation of the pulp cells with IL-1β resulted in the activation of arachidonic acid metabolism via COX-2, but not 5-LOX. Incubation with various non-toxic concentrations of the propolis extract significantly inhibited upregulated COX-2 mRNA and protein expressions upon treatment with IL-1β (p<0.05), resulting in a significant decrease in elevated PGE2 levels (p<0.05). Nuclear translocation of the p50 and the p65 subunits of NF-kB upon treatment with IL-1β was also blocked by incubation with the extract.

**Conclusions:**

Upregulated COX-2 expression and enhanced PGE2 synthesis upon treatment with IL-1β in human dental pulp cells were suppressed by incubation with non-toxic doses of Thai propolis extract via involvement of the NF-kB activation. This extract could be therapeutically used as a pulp capping material due to its anti-inflammatory properties.

## Introduction

Dental pulp infection is a common event, often found in deep dental caries and complicated crown fractures that allow microbial invasion into the pulp via direct exposure to the external milieu.^1^ Persistent stimulation of dental pulp cells with cariogenic microorganisms and pro-inflammatory mediators, released upon host-microbial interactions, poses a high risk for pulp inflammation, which is known as irreversible pulpitis.^2^ Odontoblasts and other pulp cells play a defensive role against microbial invasion via production of pro-inflammatory cytokines, particularly interleukin (IL)-1β.^3^ An increase in IL-1β synthesis has been shown to upregulate expressions of IL-8, intercellular adhesion molecule-1, and vascular adhesion molecule-1, attracting neutrophils to the infected pulp.^4 , 5^ In addition, the permeability of pulpal vasculature is increased by an upregulated expression of cyclooxygenase-2 (COX-2) and production of prostaglandin E2 (PGE2), an important arachidonic acid metabolite involved in pulpal pain. Accordingly, previous studies have demonstrated a significant elevation of PGE2 levels as the pulp tissue shifts toward symptomatic pulpitis in both deciduous and permanent molars.^6 , 7^

In case of a deep cavity, if the pulp is diagnosed as reversible pulpitis, dental lining materials are recommended for pulp protection to maintain its healthy status. Many studies have suggested the use of calcium hydroxide paste and mineral trioxide aggregate (MTA) due to their beneficial effects on promotion of pulpal wound healing and induction of reparative dentine formation.^8 - 10^ Other lining agents have later been studied for use in pulp capping, such as propolis and bee glue. The use of propolis as a pulp capping agent may hold therapeutic purposes due to its reported antimicrobial and anti-inflammatory properties^11^ that could possibly enhance the pulpal healing process. Accordingly, a study demonstrated that the exposed pulp in rats *’* teeth, when capped with propolis flavonoids, exhibited mild inflammation in the pulp tissue with reparative dentine formation by the fourth week.^12^ Moreover, the magnitude of inflammatory cell infiltration and dentine bridge formation at the exposure site of human pulp lined with propolis extract were found to be comparable to those lined with Dycal.^13^

Our previous studies have revealed the potential therapeutic benefits of Thai propolis extract as an alternative storage medium for avulsed teeth. The viability of periodontal ligament (PDL) cells in experimentally avulsed premolars was preserved by the propolis extract, similar to Hank’s balanced salt solution and milk;^14^ additionally, mRNA expression of periostin in PDL cells was also maintained by the extract up to 12 h.^15^ Regarding another study, this extract could promote pulpal wound healing with little inflammation and formation of dentine bridge with well-organized dentinal tubules in partial pulpotomy of rabbits’ teeth.^16^ Therefore, Thai propolis extract is biocompatible with both PDL and pulp cells, in addition to possibly exert an anti-inflammatory activity, promoting pulpal wound healing. Although an earlier study demonstrated an anti-inflammatory effect of Indonesian propolis against COX-2 expression in rat dental pulp cells *in vivo* ,^17^ it has not been shown whether Thai propolis also exerts its anti-inflammatory action in human dental pulp cells via suppression of COX-2/PGE2 induction upon treatment with a pro-inflammatory cytokine, IL-1β, or whether the 5-LOX pathway is involved in the anti-inflammatory effect in these cells. Therefore, this study aimed to examine the anti-inflammatory effect of Thai propolis extract on the arachidonic acid pathway activated by IL-1β in cultured human dental pulp cells, in addition to elucidate the signaling molecules mediating this effect.

## Methodology

### Preparation of Thai propolis extract

Thai propolis extract was prepared as described elsewhere.^14^ Briefly, propolis from Nong Khai province of Thailand was extracted with 95٪ ethanol, lyophilized, and then reconstituted in dimethyl sulfoxide (DMSO), which was used as a solvent to make a master stock at 1,000 mg/ml. This stock solution was kept in a bottle wrapped with aluminum foil and stored at 4°C prior to experimentation.

### Culture of primary human dental pulp cells

The study protocol was approved by the office of the Khon Kaen University Ethics Committee in Human Research, in accordance with the Declaration of Helsinki, as revised in 2013 (Approval number: HE622246). Normal pulp tissues were obtained from three upper or lower nonfunctional third molars (n=3), freshly extracted from three healthy volunteers (18–25 years old), who underwent a simple exodontia at the Department of Oral and Maxillofacial Surgery, Faculty of Dentistry, Khon Kaen University. These teeth exhibited no evidence of carious lesions, cracks, restorations, or involvement with periodontal disease. Informed consent form was obtained from all donors participating in the study. Immediately after extraction, all teeth were stored in the growth medium, *i.e.* , Dulbecco’s modified Eagle’s medium (DMEM; Gibco^®^, by Life Technologies Corporation, Carlsbad, CA, USA), supplemented with 10٪ heat-inactivated fetal bovine serum (FBS), 100 units/ml penicillin, 100 µg/ml streptomycin, and 25 µg/ml amphotericin B. Each tooth crown was circumferentially separated using a carborundum disc and an extraction forceps. Pulp tissue was removed and immediately submerged in a 35-mm culture dish containing DMEM. The tissue explant was cut into several pieces using a scalpel blade #15 and cultured in the growth medium at 37°C under 5٪ CO_2_ atmosphere with 95٪ relative humidity. The growth medium was replenished every other day. At 80٪ cell confluence, primary dental pulp cells overgrown from the explant were trypsinized to further expand their number, and the pulp cells at the third to the eighth passages were used for further experiments. In this study, the experiments mentioned were performed in triplicate for three independent repeats using three different primary human dental pulp cell lines that were cultured separately.

### Flow cytometry

To characterize cultured human dental pulp cells, the cells were trypsinized, washed with 1X Dulbecco’s phosphate-buffered saline (PBS; Gibco^®^, by Life Technologies Corporation) twice, and reacted with the following conjugated antibodies (10 µl of each antibody for 1x10^6^ cells), including antibodies against human CD34-FITC, CD45-Kro, CD105-PE (Beckman Coulter, Inc., Brea, CA, USA), CD73-Pacific Blue, and CD90-APC (BioLegend, San Diego, CA, USA) for 45 min at room temperature in the dark. The isotype of these five antibodies against human cell surface CD markers is IgG1. Furthermore, human peripheral blood mononuclear cells (hPBMC) were isolated from a whole blood sample obtained from a healthy donor, using Ficoll-Paque^TM^ density gradient centrifugation (GE Healthcare BioSciences, Uppsala, Sweden), as previously described.^18^ Then, hPBMC from the buffy layer were transferred into a 50-ml tube, washed with PBS twice, and centrifuged at 2,000 rpm for 5 min. The supernatant was discarded, and the cell pellet was resuspended in the staining buffer for the final cell density at 1x10^7^ cells/ml. The hPBMC at 1x10^6^ cells were reacted with 10 µl of each aforementioned antibody. After being washed with PBS, the stained cells were resuspended with 500 µl of sheath fluid, and at least 10,000 stained cells were acquired and analyzed using a CytoFLEX S flow cytometer (Beckman Coulter, Inc.). The unstained cells served as a negative control.

### Cytotoxic analysis of Thai propolis extract

The PrestoBlue cytotoxic assay was used to determine non-toxic concentrations of Thai propolis extract for cultured dental pulp cells, as previously described.^15^ In brief, the pulp cells were seeded into a 96-well plate (Corning Incorporated, NY, USA) and cultured in the growth medium for 24 h. On the following day, the cells were treated with the extract at 5, 2.5, 1.25, 0.63, 0.31, 0.16, 0.08, 0.04, 0.02, or 0.01 mg/ml, using serum-free DMEM to serially dilute by two-fold. The final concentration of DMSO in the extract did not surpass 0.1٪ (vol/vol). Cells treated with 10٪ (vol/vol) DMSO served as positive control due to its cytotoxicity via induction of cell apoptosis,^19^ whereas untreated cells served as negative control. After 48 h, culture supernatant was removed, and the cells were washed twice with PBS and incubated with a 50-µl volume of the PrestoBlue reagent (Gibco^®^, by Life Technologies Corporation) per well for 60 min. The fluorescence activity was measured at 560/590-nm wavelengths using a microplate reader (Varioskan Flash, Thermo Fisher Science, Vanntaa, Finland). The percentage of cell viability was calculated using the equation: (z-x/y-x) x 100, where x, y, and z are the fluorescence values of negative control, positive control, and experimental samples, respectively. Using the cells isolated from three different donors, three independent experiments were performed in triplicate, and a mean percentage of the cell viability was computed. Non-toxic concentrations of the diluted Thai propolis extract were further used to test its inhibitory effect on IL-1β-stimulated human dental pulp cells.

### Treatment with IL-1β and Thai propolis extract

Cultured dental pulp cells were first pretreated with the exogenously added human recombinant IL-1β (R&D Systems, Inc., Minneapolis, MN, USA) at 10 ng/ml for 24 h to mimic the clinical scenario, in which the dental pulp cells are at the inflammatory state prior to application of pulp capping agents. Furthermore, COX-2 expression was clearly upregulated upon treatment with IL-1βα or IL-1β for a long period of incubation.^20 , 21^ The growth medium was then removed, and pulp cells were washed twice with sterile PBS. Afterwards, cells were cultured in the fresh growth medium, supplemented with IL-1β at 10 ng/ml in the presence or absence of the non-toxic concentrations of Thai propolis extract for additional 48 h. Our preliminary findings showed that suppression of the COX-2 expression by the extract at a 48-h incubation period was more pronounced than that at a 24-h incubation period (data not shown). Therefore, the 48-h incubation with both IL-1β and Thai propolis extract was applied for all experiments. The pulp cells, treated with 10 µM of indomethacin (Sigma-Aldrich, St. Louis, MO, USA) or cultured in the growth medium only, served as a positive or a negative control, respectively.

### RNA isolation and RT-qPCR

Total RNA was extracted using the RNA and protein purification kit (NucleoSpin^®^ RNA/Protein, MACHEREY-NAGEL GmbH & Co. KG, Dueren, Germany) according to the manufacturer’s protocol. An RT-qPCR analysis was performed using a two-step procedure, in which complementary DNA (cDNA) was first synthesized from a 500-ng amount of total RNA using the High-Capacity cDNA Reverse Transcription kit (Thermo Fisher Scientific, Carlsbad, CA, USA). A 20-µl volume of qPCR contained 5٪ (vol/vol) cDNA, 10 µl of the SYBR Green master mix (PowerUp SYBR Green Master Mix, Applied Biosystems by Thermo Fisher Scientific), and 10 µM of each pair of oligonucleotide primers for COX-2^21^ (forward, TTCAAATGAGATTGTGGGAAAAT; reverse, AGATGNATCTCTGCCTGAGTATCTT), 5-lipoxygenase^22^ (5-LOX; forward, AAAGAACTGGAAACACGTCAGAAA; reverse, AACTGGTGTGTACAGGGGTCAGTT), or glyceraldehyde 3-phosphate dehydrogenase^15^ (GAPDH; forward, ACCACAGTCCATGCCATCACTGC; reverse, TCCACCACCCTGTTGCTGTAGC). The reaction, performed in the LightCycler^®^ 480 instrument (Roche Molecular Biochemicals, Mannheim, Germany), consisted of denaturation at 95°C 20 sec, annealing at 60°C 20 sec, and extension at 72°C 10 sec, for 40 cycles. GAPDH was used as a housekeeping gene control to normalize the degree of COX-2 or 5-LOX mRNA expression in each sample. Three independent assays using three dental pulp cell lines derived from three different donors were performed in triplicate. Using the ΔΔCt method, the mRNA expression of COX-2 or 5-LOX in each experimental sample was calculated in relation to that in an untreated control, set to 1.

### Immunoblot analysis

Total protein was extracted from both treated and untreated dental pulp cells using the RIPA buffer (Thermo Fisher Scientific). Cell lysate was sonicated on ice for 12 min. The concentration of total protein was determined using the Bio-Rad protein assay kit (Bio-Rad Laboratories, Inc., Hercules, CA, USA). A 10-µg amount of total protein in each sample was resolved on 12٪ SDS-PAGE and transferred to 0.45-µm nitrocellulose membrane (Amersham Protran^®^ Premium Western blotting membranes, GE Healthcare UK Limited, Buckinghamshire, UK). The membrane was subsequently blocked with 5٪ skim milk, containing 0.05٪ Tween-20 for 1 h at room temperature, and then probed with the mouse anti-human COX-2 (1:1000; Santa Cruz Biotechnology, Santa Cruz, CA, USA) or the mouse anti-human β-actin (1:1000; Santa Cruz Biotechnology) antibody for 2 h at room temperature. After being washed with PBS, containing 0.05٪ Tween-20 for 5 min, the membrane was incubated with the sheep anti-mouse IgG conjugated with horseradish peroxidase (1:1000; GE Healthcare UK Limited) for 2 h. The band signals on the membrane were detected by the Enhanced Chemiluminescence system (Amersham ECL Western Blotting Detection reagents, GE Healthcare UK Limited) and captured by the Gel Documentation system (G:BOX Chemi XL, Syngene, Cambridge, UK). Densitometric analysis was performed using ImageJ (NIH, Bethesda, MD, USA) to quantify the protein expression of COX-2, normalized by β-actin expression, in each experimental sample relative to that in an untreated control, set to 1.

### PGE2 ELISA

Conditioned media were collected and quantified for the levels of secreted PGE2 by a competitive ELISA, as previously described.^21^ Briefly, a 150-µl of culture supernatant, mixed with 50 µl of mouse monoclonal antibody to human PGE2, was added into each well of a 96-well plate, pre-coated with the goat anti-mouse polyclonal antibody (KGE004B; Prostaglandin E2 Parameter Assay Kit, R&D Systems, Inc.) for 1 h at room temperature. After washing, PGE2 conjugated with horseradish peroxidase was added in the plate for 1 h. The reaction was developed by incubation with tetramethylbenzidine as a substrate for 30 min. The optical density of each reaction was determined at 540/450-nm wavelengths, and the concentration of secreted PGE2 in each sample was derived by comparing the average absorbances of the known PGE2 concentrations.

### Immunofluorescence

Human dental pulp cells were seeded in Lab-Tek chamber slides (Thermo Fisher Scientific) and cultured in the growth medium in the presence or absence of IL-1b at 10 ng/ml with or without 0.08 mg/ml of Thai propolis extract for 1 h. Immunofluorescence was conducted as previously described^23^ to localize the presence of the p50 and the p65 subunits of nuclear factor-kappaB (NF-kB) in the cell nuclei. Briefly, the cells were fixed with 4٪ paraformaldehyde in PBS at 4°C for 10 min, permeabilized with 0.05٪ Triton X-100 (Amresco, Solon, OH, USA), and blocked with 3٪ bovine serum albumin (Sigma-Aldrich, St.Louis, MO, USA) in PBS. The cells were then reacted with the mouse monoclonal antibody to the p50 or to the p65 subunit (1:200; Santa Cruz Biotechnology) for 1 h, followed by reaction with the donkey anti-mouse IgG conjugated with NorthernLight557 (1:500; R&D Systems, Inc.), and 4’,6-diamidino-2-phenylindole (DAPI; 1:500; Biotium, Inc., Hayward, CA, USA) for 1 h. The presence of nuclear localization of the p50 and the p65 subunits of NF-kB was visualized by a fluorescence microscope (AxioImage Z2m, Carl Zeiss Microscopy GmbH, Göttingen, Germany), and their images were captured by an attached digital camera. The percentages of nuclear staining for both p50 and p65 subunits of NF-kB from the captured images were computed and compared between different groups.

### Statistical analysis

All data are presented as mean ± standard deviation. Due to their normal distribution, as determined by Shapiro-Wilk test, One-way ANOVA and Bonferroni’s post hoc comparison test via SPSS^®^ version 21 (IBM, Chicago, IL, USA) were used to assess differences in the mean percentages of cell viability, degrees of COX-2 mRNA and protein expressions, PGE2 levels, and percentages of nuclear staining of NF-kB . The differences were considered statistically significant at the *p* -value<0.05.

## Results

**Figure 1:**
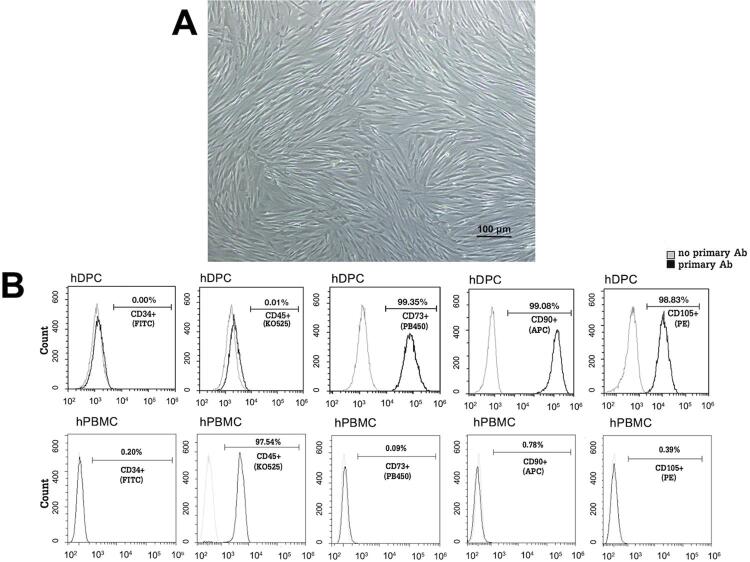
Characterization of human dental pulp cells (hDPC). (A) The fibroblast-like morphology of cultured hDPC, isolated from pulp tissue explants. This image was taken at the 50X magnification power. Scale bar = 100 μm. (B) By flow cytometry, expressions of three mesenchymal stromal cell markers, including CD73 (PB450), CD90 (APC), and CD105 (PE) were found in hDPC, whereas expression of CD34 (FITC) and CD45 (KO525) was not found. Results are representative of three different dental pulp cell lines from three healthy donors with the same finding. On the contrary, expression of CD45 (KO525) was only detected in human peripheral blood mononuclear cells (hPBMC)

Due to heterogeneous cell populations found within the dental pulp, cultured human dental pulp cells were morphologically and molecularly characterized. The spindle fibroblast-like cell shape was observed for most of these cultured cells ( Figure 1A ). Consistently, approximately 99٪ of these cells were found to express three classical mesenchymal stromal cell (MSC) surface markers, including CD73, CD90, and CD105, whereas expression of CD34, a marker of hematopoietic and endothelial stem cells, or that of CD45, a marker of white blood cells, was virtually absent ( Figure 1B ), indicating an MSC type of the cultured human dental pulp cells, as previously defined.^24^ In contrast to human dental pulp cells, almost all hPBMC (97.54٪) were positively stained for CD45, while they were virtually unstained for the other three mesenchymal CD markers or CD34 ( Figure 1B ). Since the isotype of all five conjugated antibodies against cell surface CD markers used in this study is the same, i.e., IgG1, the negative staining with anti-CD45 antibody in human dental pulp cells can, thus, be regarded as an isotype antibody control for the positive staining with anti-CD73, anti-CD90, and anti-CD105 antibodies in the same cell type. Note that the anti-CD45 antibody was still found to be effective for staining hPBMC.

**Figure 2:**
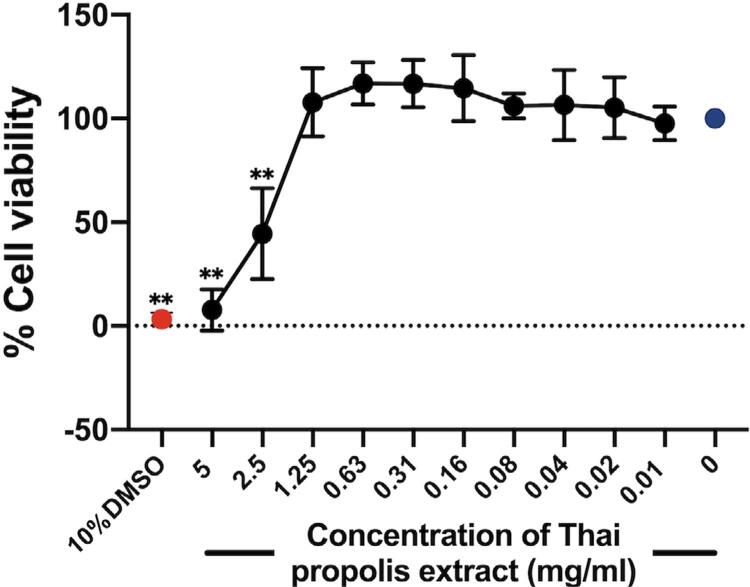
Determination of the cytotoxicity of Thai propolis extract by the PrestoBlue assay. Cultured human dental pulp cells were treated with indicated doses (0.01-5 mg/ml) of the extract for 48 h. The line graph demonstrates the average percentages of cell viability from three independent experiments, each of which was performed in triplicate, using three different dental pulp cell lines. Error bars = standard deviation; **p<0.01, as compared with the percentage of cell viability in an untreated control, set to 100 (blue circle). 10% DMSO (red circle) = treatment with 10% (vol/vol) of dimethyl sulfoxide for 48 h

By the PrestoBlue cytotoxic assay, the mean percentages of cell viability upon a 48-h treatment with various concentrations of Thai propolis extract up to 1.25 mg/ml were not found to be significantly different from that of untreated cells, whereas treatment with the extract at 2.5 or 5 mg/ml significantly decreased the mean percentages of cell viability, indicating the cytotoxicity of these two doses ( *p* <0.01; Figure 2 ). As a control, treatment with 10٪ (vol/vol) DMSO for 48 h was very toxic to the cells ( Figure 2 ). Therefore, various concentrations of Thai propolis extract up to 1.25 mg/ml were chosen for further experiments.

### Induction of COX-2, but not 5-LOX, by IL-1β treatment in human dental pulp cells

**Figure 3:**
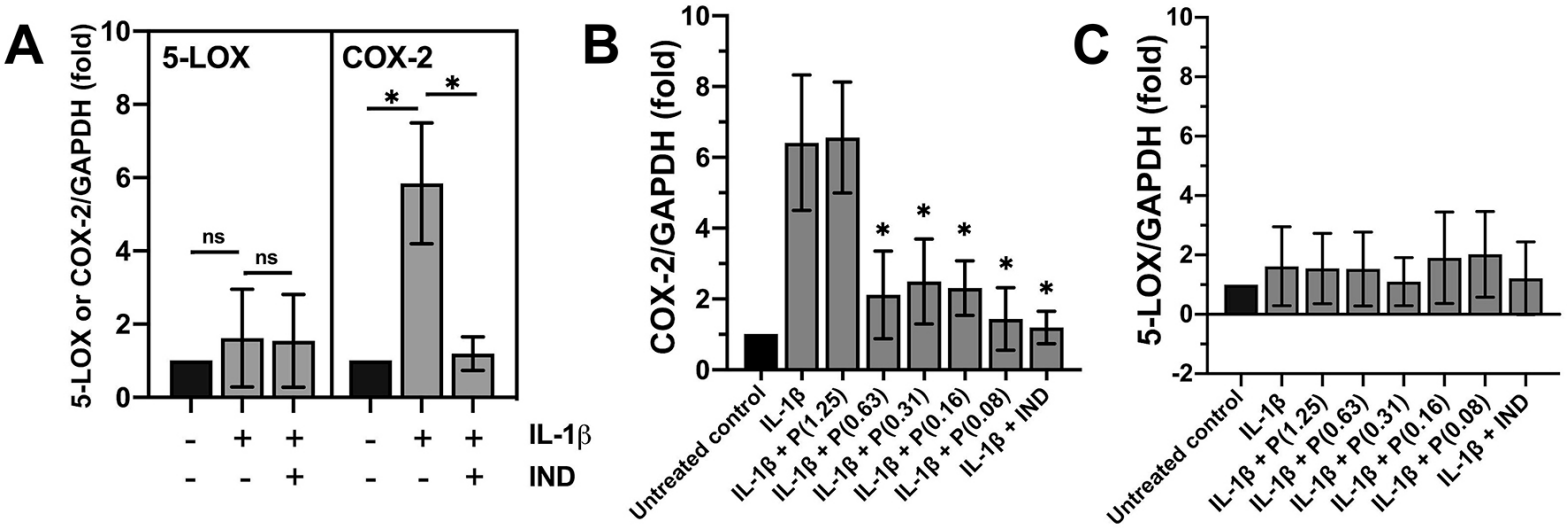
IL-1b mediates signaling via COX-2 in human dental pulp cells. (A) Significant induction of COX-2 mRNA, but not 5-LOX mRNA, upon IL-1b treatment in human dental pulp cells. (B) Significant inhibition of COX-2 mRNA induction upon IL-1b treatment by Thai propolis extract (P) at the doses of 0.08, 0.16, 0.31, and 0.63 mg/ml. (C) No significant difference between treatment with IL-1b at 10 ng/ml or with the extract (P) at all tested concentrations and an untreated control in the average degrees of 5-LOX mRNA expression. The three bar graphs demonstrate the mean degrees of COX-2 or 5-LOX mRNA expression from three separate experiments using three different dental pulp cell lines. Error bars = standard deviation; *p<0.05 in (A) and (B), as compared with the mean degree of COX-2 mRNA induction in the pulp cells treated with IL-1b at 10 ng/ml; ns = not significant; IND = treatment with indomethacin at 10 µM

Upregulated expressions of COX-2 and 5-LOX, two major enzymes used to synthesize prostanoids in the arachidonic acid pathway, were first determined according to their involvement in the inflammation of dental pulp cells upon IL-1b treatment. By RT-qPCR, treatment with IL-1b at 10 ng/ml in the dental pulp cells significantly induced mRNA expression of COX-2 by approximately six-fold when compared with an untreated control ( *p* <0.05). However, mRNA expression of 5-LOX was not significantly upregulated by IL-1b treatment ( Figure 3A ), suggesting that human dental pulp cells selectively utilize COX-2, but not 5-LOX, to catalyze the arachidonic acid metabolism to synthesize the prostanoids in response to IL-1b stimulation. The importance of COX-2 in IL-1b-induced inflammation in the dental pulp cells was also confirmed by a significant inhibition of upregulated COX-2 mRNA expression ( *p* <0.05), but not 5-LOX mRNA expression, by treatment with 10 µM of indomethacin, a non-steroidal anti-inflammatory drug ( Figure 3A ).

### Inhibition of COX-2 induction and PGE2 production by Thai propolis extract

To determine the inhibitory effect of Thai propolis extract on the inflammation, human dental pulp cells were treated with IL-1b at 10 ng/ml in the presence or absence of various non-toxic doses (0.08–1.25 mg/ml) of the extract for 48 h. By RT-qPCR, induction of COX-2 mRNA expression upon IL-1b treatment was significantly inhibited by treatment with the extract at the doses from 0.08 to 0.63 mg/ml ( *p* <0.05; Figure 3B ), whereas the extract at any doses tested in this experiment failed to inhibit 5-LOX mRNA expression ( Figure 3C ). Notably, upregulated COX-2 mRNA expression upon IL-1b treatment was not blocked by treatment with the extract at 1.25 mg/ml ( Figure 3B ). As with the inhibitory effect of Thai propolis extract, induction of COX-2 mRNA expression, but not 5-LOX mRNA expression, upon IL-1b treatment was significantly abrogated by treatment with indomethacin at 10 µM ( *p* <0.05; Figure 3B and C ).

**Figure 4:**
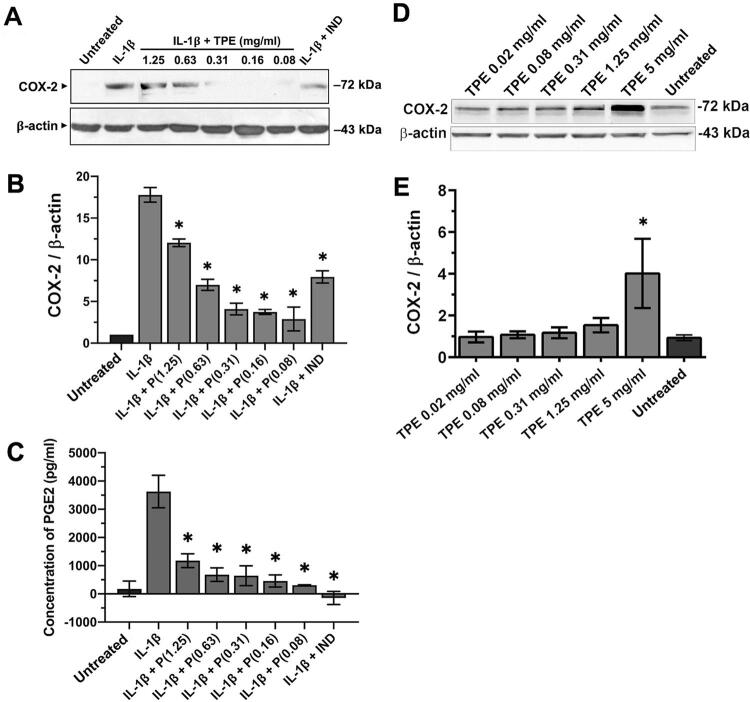
Inhibition of COX-2 protein induction and raised PGE2 levels upon treatment with IL-1b at 10 ng/ml by Thai propolis extract in human dental pulp cells. (A) A representative immunoblot from three independent experiments using three different dental pulp cell lines with a similar finding, showing the absence of COX-2 band, detected at 72 kDa, by treatment with the extract (TPE) at 0.08 and 0.16 mg/ml. (B) Significant inhibition of the mean degrees of COX-2 protein expression by the extract (P) at all doses tested, as quantified by densitometry from protein bands in (A). (C) Significant decreases in the mean concentrations of PGE2 in pg/ml, released into conditioned media of the samples in (A). *p<0.05 in (B) and (C), as compared with the mean degree of COX-2 protein induction and the mean level of elevated PGE2, respectively, in the cells treated with IL-1b at 10 ng/ml; IND = treatment with indomethacin at 10 µM. (D) Immunoblots showing the effects of treatment with various doses of Thai propolis extract (TPE) alone on COX-2 protein expression and its densitometry (E). *p<0.05, a significant increase in the mean degree of COX-2 protein expression, as compared with those of other samples. Error bars in (B), (C), and (E) = standard deviation

By immunoblot analysis, induction of COX-2 protein, detected at 72 kDa upon IL-1b treatment, was evidently inhibited by the Thai propolis extract at the doses from 0.08 to 0.31 mg/ml ( Figure 4A ). Protein expression of b-actin, used as a housekeeping gene control, was found to be equivalent among different samples. By densitometry, the inhibition of COX-2 protein induction upon IL-1b treatment was found to be significant at the extract doses ranging from 0.08 to 1.25 mg/ml ( *p* <0.05; Figure 4B ), which was confirmed by significant decreases in elevated PGE2 levels upon IL-1b stimulation by treatment with the extract at the doses from 0.08 to 1.25 mg/ml ( *p* <0.05; Figure 4C ). Note that an inverse dose-dependent inhibition of COX-2 protein expression and PGE2 levels was found ( Figure 4B and C ). As a positive control, treatment with indomethacin at 10 µM significantly inhibited COX-2 protein induction and, thus, reduced PGE2 levels upon IL-1b treatment ( *p* <0.05; Figure 4B and C , respectively). When human dental pulp cells were incubated with various doses of the extract alone, a slight increase in COX-2 protein expression upon treatment was observed by the 1.25 mg/ml dose ( Figure 4D and E ), probably as a result of mild cytotoxicity that may have accounted for a lower reduction in upregulated COX-2 protein expression ( Figure 4B ) and in raised PGE2 levels ( Figure 4C ) by this dose than a remarkable COX-2/PGE2 decrease by the low doses at µg/ml. Interestingly, the extract at 5 mg/ml that was found to be cytotoxic ( Figure 2 ) upregulated, substantially and significantly, COX-2 protein expression ( *p* <0.05; Figure 4D and E ). Thus, the inhibitory effects against COX-2 mRNA and protein inductions as well as against enhanced PGE2 synthesis in response to IL-1b treatment in cultured human dental pulp cells seemed to be more pronounced by Thai propolis extract at lower doses ( Figure 3 and 4 ).

### Involvement of the p50 and the p65 subunits of NF-kB in the anti-inflammatory effect of Thai propolis extract

**Figure 5:**
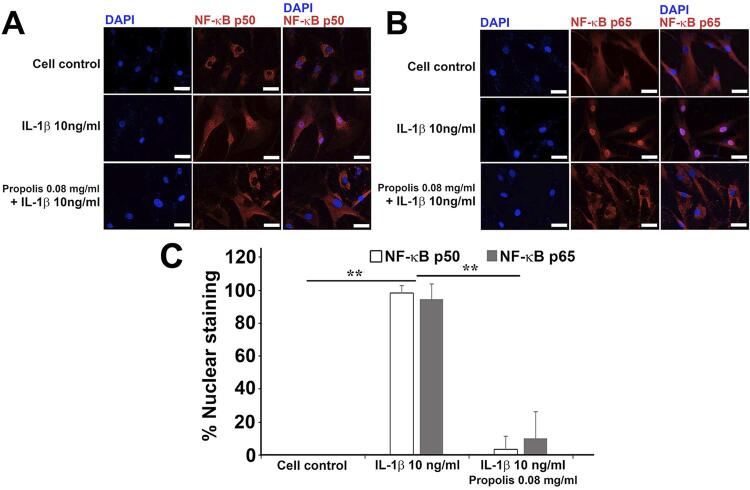
Representative immunofluorescence images from three independent experiments using three different human dental pulp cell lines. Immunoreaction with the mouse monoclonal antibody to the p50 (A) or to the p65 (B) subunit of NF-kB, followed by reaction with the anti-mouse IgG conjugated with NorthernLight557 (red) demonstrates the localization of each subunit. DAPI staining (blue) indicates the location of nuclei. Scale bars = 50 μm. (C) A quantitative analysis for the percentages of nuclear staining of both p50 and p65 subunits from (A) and (B), respectively. **p<0.01, as compared with the mean percentages of nuclear staining for both p50 and p65 subunits in the IL-1b-treated sample

Because induction of COX-2 expression upon IL-1β treatment was demonstrated to involve NF-kB activation in human gingival fibroblasts,^25^ nuclear translocation of the p50 and the p65 subunits of NF-kB was, therefore, examined in human dental pulp cells. In addition, since the most effective dose of Thai propolis extract that inhibits COX-2 and PGE2 was found to be at 0.08 mg/ml ( Figure 4 ), this dose was only selected for an immunofluorescence analysis. Both p50 and p65 subunits were found to be localized in the cytoplasm of control untreated dental pulp cells, but they were translocated into the nuclei of these cells upon treatment with IL-1b at 10 ng/ml ( Figure 5A and B ). Co-incubation with 0.08 mg/ml of the extract shown to inhibit both COX-2 mRNA and protein inductions, in addition to elevate PGE2 levels ( Figure 3 and 4) and block nuclear translocation of both subunits ( Figure 5A and B ). By a quantitative analysis, a significant inhibition for the percentages of nuclear translocation of both subunits was found by treatment with the extract at 0.08 mg/ml ( *p* <0.01; Figure 5C ), suggesting that the extract is likely to exert its inhibitory effect on COX-2 induction and enhanced PGE2 synthesis upon IL-1b treatment via blockade of nuclear translocation of NF-kB.

## Discussion

Pulpal inflammation and pain are major challenges in Endodontics, for which mediation of the COX-2/PGE_2_ pathway is reported to be responsible.^26 , 27^ In this study, the non-toxic doses of Thai propolis extract were investigated for its anti-inflammatory effect against COX-2/PGE2 induction upon treatment with IL-1β in cultured human dental pulp cells. Our results showed that the expression of COX-2, but not 5-LOX, was upregulated in response to treatment with IL-1β in cultured human dental pulp cells, resulting in elevated PGE2 levels, suppressed upregulation of COX-2 and PGE2 in IL-1β-treated human dental pulp cells, and blocked nuclear translocation of both p50 and p65 subunits of NF-kB, suggesting that Thai propolis extract is likely to target both pulpal inflammation and pain via the COX-2/PGE2 pathway and NF-kB signaling.

Vital pulp therapy aims to maintain pulp vitality by eliminating bacteria from the dentine-pulp complex, and to establish an environment to promote pulpal wound healing. Some materials have been used for vital pulp treatment, including the traditional material of choice, calcium hydroxide, and hydraulic calcium silicate cements, *i.e.* , MTA.^10^ A recent study has suggested that Thai propolis extract could be another promising alternative material used for pulp capping procedures because its application on the mechanically exposed pulp induces the formation of reparative dentine with orderly arranged dentinal tubules.^16^ In addition to its reparative dentine induction^16^ and anti-inflammatory properties shown in this study, the Thai propolis extract has antimicrobial properties against the growth of *Streptococcus* and *Lactobacillus* species,^28 , 29^ making this extract a good candidate material for vital pulp therapy.

After treatment of human dental pulp cells with IL-1β, mostly secreted by odontoblasts and dental pulp cells in response to cariogenic infection,^3 , 30^ a significant induction of COX-2, but not 5-LOX, was found, suggesting that the 5-LOX pathway of arachidonic acid metabolism is not involved in an IL-1β-mediated inflammatory pulpal response. This finding agrees with previous *ex vivo* studies, showing that COX-2 expression was considerably induced in inflamed dental pulp tissues, in which it was found to be at the dental pulp fibroblasts.^7 , 31^ Upon treatment with Thai propolis extract in human dental pulp cells treated with IL-1β, the PGE2, a potent inflammatory mediator generated by COX-2, was significantly decreased, proposing that the inhibitory effect of the extract against inflammatory responses in human dental pulp cells is via the COX-2/PGE2 pathway.

Since the propolis extract contains a cocktail of natural flavonoids, the immunoregulatory effect of Thai propolis extract on human dental pulp cells is presumably driven by these flavonoids, such as flavonols and flavones,^32 , 33^ which were previously demonstrated to suppress prostaglandin endoperoxide synthase.^34^ Other active compounds that may be present in the Thai propolis extract can also play a pivotal role in controlling the anti-inflammatory activity. Caffeic acid phenethyl ester (CAPE), another major constituent of propolis, is a potent modulator of the arachidonic acid metabolism because it prevents the release of arachidonic acid from the cell membrane and inhibits gene expressions of LOX and COX.^34^ It also inhibits the nuclear translocation of NF-kB, preventing its activation that ultimately leads to COX-2 downregulation in human leukocytes and T cells.^35 , 36^ The inhibitory effect of CAPE against NF-ΚB activation is consistent with the blockade of IL-1β–mediated nuclear translocation of both p50 and p65 subunits of NF-kB by the Thai propolis extract shown in this study. Thus, the suppression of COX-2/PGE2 pathway and NF-kB signaling may be due to the presence of CAPE in Thai propolis extract. A comprehensive characterization of active compounds in the Thai propolis extract along with validation of their biological properties would refine our insights into the immunomodulatory effects of Thai propolis in human dental pulp cells.

Our current study was conducted using non-toxic doses of Thai propolis extract, ranging from 0.08 to 1.25 mg/ml. It was found that upregulated COX-2 expression and raised PGE2 levels in response to the treatment with IL-1β were remarkably dampened by the extract, although the inhibition of COX-2 and PGE2 induction by the extract at 1.25 mg/ml was not as pronounced at lower concentrations, possibly due to mild cytotoxicity. It is noteworthy that the cytotoxic dose of Thai propolis extract at 5 mg/ml considerably induced COX-2 protein expression, which is consistent with the cytotoxicity of dental restorative monomers accompanied by COX-2 induction observed in human dental pulp cells.^37 , 38^

Even though previous investigations have addressed a potential use of propolis as a pulp capping material,^12 , 13^ formulation of the propolis extract to improve its physical characteristics is needed for more practical clinical applications with sustained therapeutic effects and a longer shelf life than its sole active chemical. A previous study has revealed that the release of propolis extract incorporated into polyvinyl alcohol, aimed at a prolonged release, reached its plateau level within 48 h.^39^ Correspondingly, the 48-h incubation of the Thai propolis extract at 0.08 mg/ml in close contact with cultured dental pulp cells has shown the remarkably suppressive effects on COX-2 and PGE2 induction. Given the significance of the dose- and time-dependent responses of dental pulp cells to Thai propolis extract, studies of an appropriate pharmaceutical formulation are, therefore, required to apply this extract for future clinical use as a pulp capping agent that can exert long lasting effects on dental pulp repair and reparative dentine formation.

## Conclusions

Thai propolis extract exerts an anti-inflammatory activity against COX-2 induction and enhanced PGE2 synthesis upon IL-1β treatment in human dental pulp cells. The anti-inflammatory effect of this extract is involved with blockade of nuclear translocation of the p50 and the p65 subunits of NF-kB upon IL-1β treatment. Therefore, the anti-inflammatory properties of this extract could be beneficial for therapeutic use as an alternative pulp capping material.
